# Osteoclast-derived extracellular vesicles are implicated in sensory neurons sprouting through the activation of epidermal growth factor signaling

**DOI:** 10.1186/s13578-022-00864-w

**Published:** 2022-08-14

**Authors:** Estrela Neto, Luís Leitão, José C. Mateus, Daniela M. Sousa, Cecília J. Alves, Miguel Aroso, Ana C. Monteiro, Francisco Conceição, Richard O. C. Oreffo, Jonathan West, Paulo Aguiar, Meriem Lamghari

**Affiliations:** 1 i3S - Instituto de Investigação e Inovação em Saúde, Universidade do Porto, Rua Alfredo Allen 280, 4200-135 Porto, Portugal; 2 INEB - Instituto de Engenharia Biomédica, Universidade do Porto, Rua Alfredo Allen 280, 4200-135 Porto, Portugal; 3 ICBAS - Instituto de Ciências Biomédicas Abel Salazar, Universidade do Porto, Rua de Jorge Viterbo Ferreira n.º 228, 4050-313 Porto, Portugal; 4 Centre for Human Development, Stem Cells and Regeneration, Human Development and Health, Tremona Rd, Southampton, SO16 6YD UK; 5 Institute for Life Sciences and Cancer Sciences, University of Southampton, Highfield Campus, Southampton, SO17 1BJ UK

**Keywords:** Extracellular vesicles, Sensory neurons sprouting, Epidermal growth factor receptor (EGFR/ErbB2) signaling, Neuronal electrophysiology, Osteoclast secretome, Bone pain

## Abstract

**Background:**

Different pathologies, affecting the skeletal system, were reported to display altered bone and/or cartilage innervation profiles leading to the deregulation of the tissue homeostasis. The patterning of peripheral innervation is achieved through the tissue-specific expression of attractive or repulsive axonal guidance cues in specific space and time frames. During the last decade, emerging findings attributed to the extracellular vesicles (EV) trading a central role in peripheral tissue innervation. However, to date, the contribution of EV in controlling bone innervation is totally unknown.

**Results:**

Here we show that sensory neurons outgrowth induced by the bone resorbing cells—osteoclasts—is promoted by osteoclast-derived EV. The EV induced axonal growth is achieved by targeting epidermal growth factor receptor (EGFR)/ErbB2 signaling/protein kinase C phosphorylation in sensory neurons. In addition, our data also indicate that osteoclasts promote sensory neurons electrophysiological activity reflecting a possible pathway in nerve sensitization in the bone microenvironment, however this effect is EV independent.

**Conclusions:**

Overall, these results identify a new mechanism of sensory bone innervation regulation and shed the light on the role of osteoclast-derived EV in shaping/guiding bone sensory innervation. These findings provide opportunities for exploitation of osteoclast-derived EV based strategies to prevent and/or mitigate pathological uncontrolled bone innervation.

**Supplementary Information:**

The online version contains supplementary material available at 10.1186/s13578-022-00864-w.

## Introduction

The innervation pattern is achieved by a series of chemoattractant and chemorepellent cues secreted at the peripheral tissues, guiding the axonal projections to form functional circuits [1–3]. Axonal terminals have the machinery to accurately respond to these molecules, ensuring the correct establishment of peripheral connections [2–4].

In the bone tissue, nerve terminals display an important regulatory mechanism for bone development, turnover, and regeneration [1, 4–8]. Importantly, neuro-skeletal interaction is bidirectional as bone-resident cells are acknowledged to modulate sensory neurons by promoting or inhibiting axonal growth. We have demonstrated that the differentiation of human mesenchymal stem cells to osteoblasts (bone forming cells) leads to marked impairment of their ability to promote axonal growth [1]. The mechanisms by which osteoblasts provide this nonpermissive environment for axons include paracrine-induced repulsion [stimulation of Semaphorin 3A, Wnt4, and Sonic hedgehog (Shh) expression] and loss of neurotrophic factors expression (drastic reduction of nerve growth factor (NGF) and brain-derived neurotrophic factor (BDNF) production) [1]. On the other hand, recent studies reported that osteoclasts (bone resorbing cells) are implicated, via netrin-1 signaling, in the pathological sensory innervation of the subchondral bone and endplates in inflammatory mouse models of osteoarthritis [9] and intervertebral disc degeneration [10]. Exuberant pathological nerve sprouting has been associated with pain development, mainly in cancer-related metastases [11–13].

Research examining neuronal activity reported that extracellular vesicles (EV) are an important communication route between neurons and surrounding cells/microenvironment. In the central nervous system, vesicles released from neurons and glial cells have been implicated in mediate synaptic plasticity, neuronal survival and neuroprotection [14–16]. In the peripheral nervous system, microglial-derived EV were also reported to promote synaptic refinement and instructing neurons upon inflammatory stimuli [17]. Schwann cells-derived EV, internalized by axons, enhance axonal regeneration after nerve injury [14, 18]. Moreover, neurons respond to EV derived from other cellular populations, as seen for the increased neurite growth of cortical neurons in response to mesenchymal stem cells-derived EV [19–21] and enhanced differentiation of neuroblastoma cell line upon exposure to EV from adipocyte-derived Schwann cell-like [22].

Osteoclasts secrete EV at the bone microenvironment, in physiological and pathological conditions [23], which have been associated as key players underlying the osteoclast-osteoblast communication [24–27]. Osteoclast-derived EV were shown to either enhance or block osteoblast differentiation depending on their cargo. EV containing miRNA-214 was demonstrated to downregulate alkaline phosphatase, osteocalcin and collagen type 1 alpha [25], while osteoclasts-EV carrying RANK can bind to osteoblasts surface activating transcription factor Runx2, which promotes bone formation [27].

The current study examined the outgrowth, signaling pathways activation and electrical activity of sensory nerve fibers under the effect of osteoclast-derived EV. Skeletal nerve fibers density varies with changes in skeletal diseases and increased pain is often associated with neural ingrowth [12, 28–31]. Our results elucidate novel mechanisms for explaining the peripheral nerve growth modulation by osteoclasts, essential for pursuing new targets for bone pain therapies.

## Results

### Sensory neurons outgrowth under osteoclasts effect is not mediated by neurotrophins

**Fig. 1:**
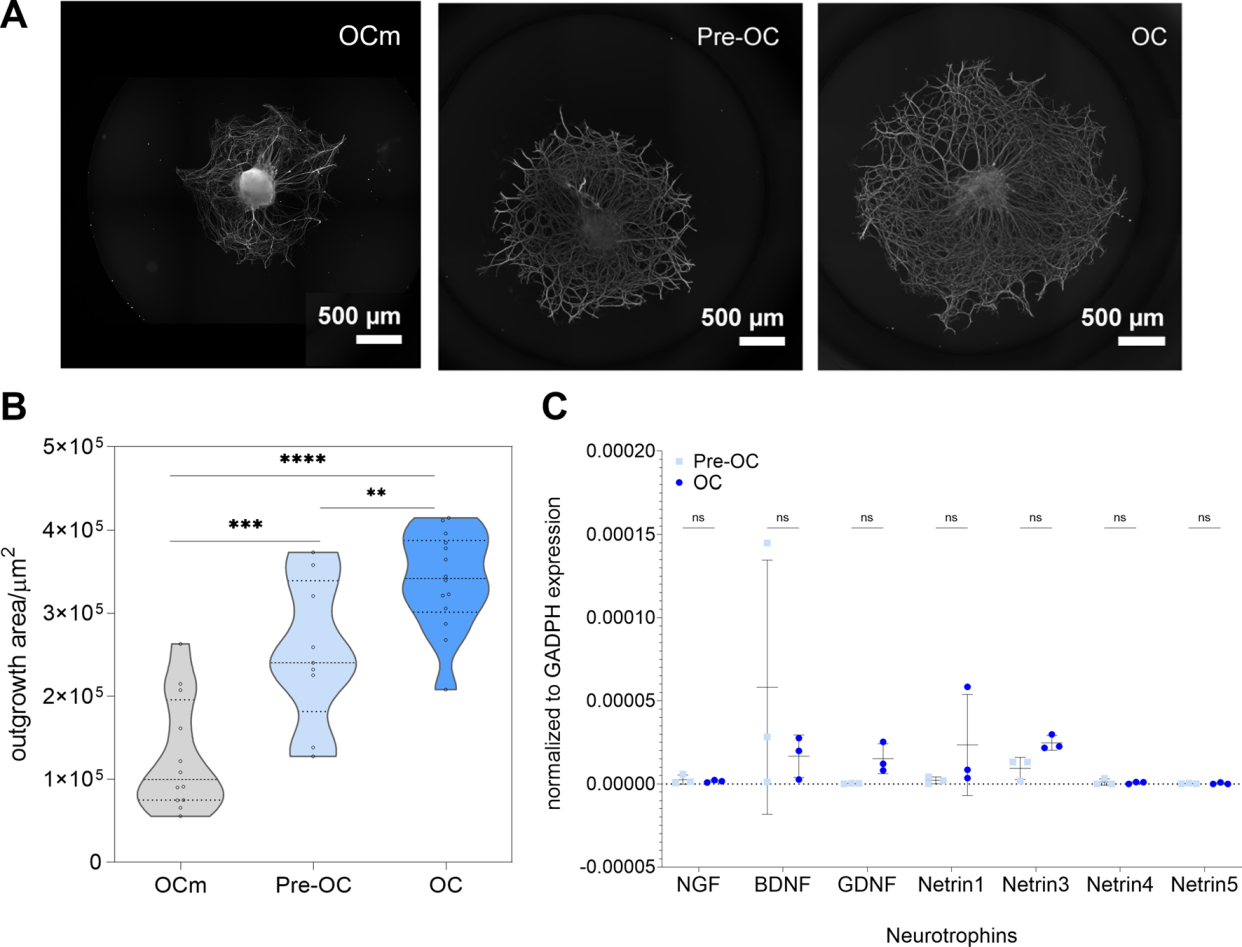
Sensory neurons axonal outgrowth is promoted by osteoclasts secretome. **A** Representative images of dorsal root ganglia (DRG) outgrowth after treatment (stained for βIII-tubulin, scale bar 500 µm). Fresh osteoclast medium [OCm: alpha-MEM supplemented with 10% fetal bovine serum (FBS), receptor activator of nuclear factor kappa-Β ligand (RANKL) and macrophage colony-stimulating factor (M-CSF)], pre-osteoclast conditioned media (Pre-OC), and mature osteoclast conditioned media (OC) were used to stimulate embryonic DRG culture. **B** Automatic axonal outgrowth area quantification. Data represented as a violin plot; **p ≤ 0.01; ***p ≤ 0.001 and ****p ≤ 0.0001. **C.** Gene expression analysis of neurotrophic factors expressed by pre-osteoclasts (Pre-OC) and mature osteoclasts (OC), normalized for the expression of glyceraldehyde 3-phosphate dehydrogenase (GAPDH) housekeeping gene. Nerve growth factor (NGF), brain-derived neurotrophic factor (BDNF), glial cell line-derived neurotrophic factor (GDNF), and netrins 1, 3, 4 and 5. Data represented as scatter dot plot mean ± SD

To explore how peripheral sensory nerves axonal growth can be modulated by the osteoclast lineage, lumbar dorsal root ganglia (DRG) were exposed to the secretome of osteoclasts at different stages of differentiation (evaluation of the osteoclast differentiation in Additional file 1: Fig. S1). Secretome from mature osteoclasts provided greater support for axonal development when compared to pre-osteoclast secretome indicating that the maturation state of osteoclasts influences the neurotrophic potential (Fig. 1A, B). The mature osteoclast secretome demonstrated an approximate threefold stronger influence on sensory neurons growth than the control of alpha-MEM medium (OCm) supplemented with receptor activator of nuclear factor kappa-Β ligand (RANKL) and macrophage colony-stimulating factor (M-CSF), cytokines described to modulate the axonal outgrowth [32, 33]. The secretome from bone marrow stromal cells (BMSC), known for its neurotrophic potential [1, 34, 35], was also evaluated and no differences were observed when compared to the NGF supplemented neurobasal and alpha-MEM controls (Additional file 1: Fig. S2).

Osteoclasts were described to induce nerve outgrowth through netrin-1 action under inflammatory conditions [9, 10]. To assess if the sensory axonal growth, induced by the osteoclast secretome under homeostatic state, was neurotrophin dependent we analyzed the expression levels of neurotrophic factors with a consolidated capability in promoting axonal growth [36]. No differences were observed between the pre-osteoclasts and osteoclasts genetic levels. All the analyzed neurotrophic factors (NGF, BDNF, GDNF, netrin-1, netrin-3, netrin-4 and netrin-5) presented low levels of genetic expression (Fig. 1C). The results were further validated by measuring the protein levels of NGF, BDNF, netrin-1, neurotrophin-3 and neurotrophin 4/5 in the conditioned medium, by enzyme-linked immunosorbent assay (ELISA) (Additional file 1: Fig. S3). Neither NGF, BDNF, NT-1 nor NT-3 were not detected in the mature osteoclast secretome, despite our observation of higher neurite outgrowth on embryonic DRG explant cultures. BDNF was detected in the pre-osteoclast conditioned medium and NT-4/5 in both conditions, still at low concentrations.

### Osteoclast-derived extracellular vesicles (EV) are directly involved in the sensory neurons axonal outgrowth

#### EV depletion from osteoclasts secretome impaired axonal growth

**Fig. 2:**
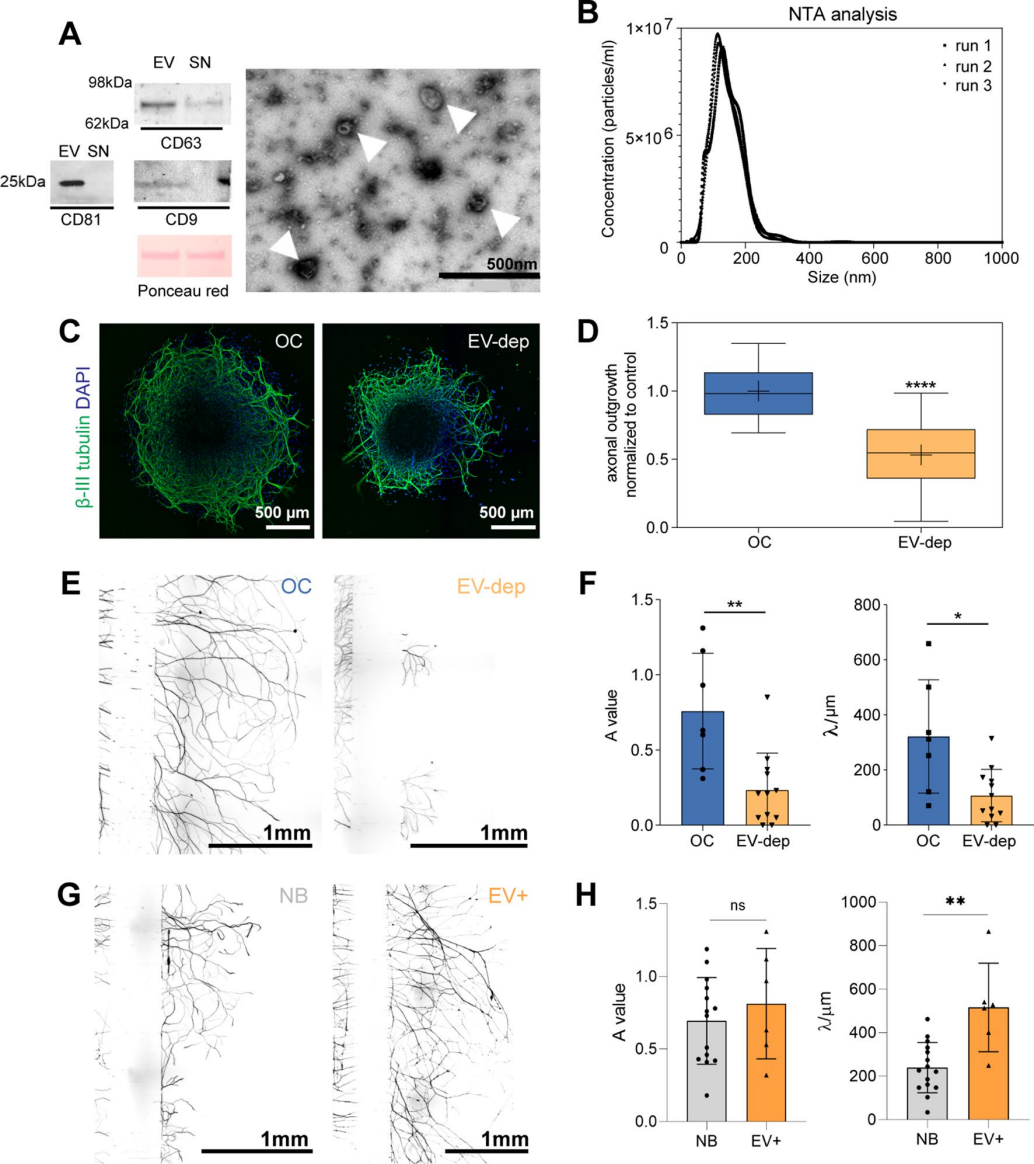
Dorsal root ganglia (DRG) axonal network growth is dependent on the osteoclasts extracellular vesicles (EV). **A** Characterization of osteoclast-derived EV by Western blot using CD81, CD63 and CD9 membrane markers [EV enriched fraction (EV) vs. EV-depleted supernatant (SN)]. Ponceau red staining showing the total amount of protein loaded. Transmission electron microscopy of osteoclast-derived EV (white arrows) by negative staining. Scale bar 500 nm. **B** Nanoparticle tracking analysis (NTA; NanoSight NS300) of the osteoclast-derived EV enriched fraction showing the concentration *vs.* size distribution (diluted in filtered PBS 1:500). Lines representing 3 runs. **C** Representative images of DRG treated with osteoclast secretome (OC) and EV-depleted osteoclast secretome (EV-dep). Staining for βIII tubulin, scale bar 500 µm. **D** Quantification of axonal sprouting area of DRG. Data represented as box and whiskers (median, whiskers represent minimum to maximum range), ****p ≤ 0.0001. **E** Representative images of DRG cultures in microfluidic devices. Nerve terminals exposed to complete osteoclasts secretome (OC) and EV-depleted osteoclasts secretome (EV-dep). Axons stained against βIII-tubulin; scale bar: 1 mm. **F** Quantification of the axonal growth using AxoFluidic algorithm. The data were given by the spatial dependence decay function $f(x) = A \cdot \exp ( - x/\lambda )$ of the axons that can effectively cross the microchannels, where the constant *A* represents the entering in the axonal compartment, and *λ* the scale of spatial decay, as a measure to represent the length of the neurites. **G** Representative images of DRG cultures in the microfluidic platforms. Nerve terminals exposed to neurobasal control (NB) and osteoclast-derived EV (EV+). Axons stained against βIII-tubulin; scale bar: 1 mm. **H** Quantification of the axonal growth using AxoFluidic algorithm. The constant *A* represents the enter in the axonal compartment, and *λ* the scale of spatial decay, as a measure to represent the length of the neurites. Results are presented as bar ± SD, ns—non-significative; *p ≤ 0.05; **p ≤ 0.01 and ***p ≤ 0.001. Each dot represents a microfluidic device analyzed from at least three independent experiments

It is increasingly appreciated that cells can release growth factors in and/or on the surface of EV/exosomes [37, 38]. We hypothesized that osteoclast-derived EV could play a crucial role in the axonal outgrowth. To test this, we exposed DRG to EV-depleted osteoclast secretome and measured axonal sprouting. The EV enriched fraction was characterized by Western Blot (WB), transmission electron microscopy (TEM) and nanoparticle tracking analysis (NTA). The EV isolated from the osteoclast secretome stained positive for the CD81, CD63 and CD9 specific markers (Fig. 2A). Cytochrome c was absent in the EV samples indicating that the EV preparations were not contaminated with cellular debris (not shown). EV were visualized by negative staining for TEM (Fig. 2A, white arrowheads), presenting a size ranging from 40 to 200 nm. The analysis of the size and concentration of the vesicles by NTA confirmed a normal distribution with a mean size of 141.8 ± 2.7 nm and a concentration of 4.90 × 10^11^ particles/mL (Fig. 2B).

DRG were exposed to EV-depleted osteoclast secretome to address the impact on axonal growth. A significant decrease in axonal sprouting was observed in the absence of EV (Fig. 2C, D). This suggests that the EV cargo plays a role in the neurotrophic potential of the osteoclast secretome. We further evaluated if the effect was equally observed at the nerve terminals. We cultured DRG in microfluidic platforms to recapitulate the in vivo state, where sensory neuron cell soma is confined to the DRG, apart from their axonal terminals in the bone microenvironment [1, 39, 40]. The reduced height of the microchannels, combined with a higher volume on the somal compartment of the microfluidic devices, creates a sustained unidirectional flow from the somal to the axonal compartment. This ensures the retention of the stimuli in the axonal compartment, therefore producing a localized effect on axon terminals. We observed that the axonal sprouting was reduced in the conditions without EV (EV-dep) when compared to the total osteoclasts secretome (OC) (Fig. 2E). Neurite outgrowth was measured with AxoFluidic, an algorithm that we designed to quantify neurite projection within microfluidic platforms [39]. The AxoFluidic calculates two major parameters: *A* represents the amount of axons that arrive at the axonal compartment and *λ* represents the scale of spatial decay (associated with the length of the neurite). Both constants (A and λ) were significantly reduced in the conditions where nerve terminals were exposed to EV-depleted secretome, when compared to the full secretome (Fig. 2F). This indicates that the lack of EV in the osteoclast secretome leads to fewer and shorter neurites in the axonal compartment.

#### Osteoclast-derived EV promote axonal growth

To evaluate the direct interaction of osteoclast-derived EV on sensory neurons, we exposed the axonal terminals of DRG, cultured in the microfluidic devices, to the EV enriched fraction isolated from the mature osteoclast secretome. Axons were exposed to a concentration of 10^11^ EV/mL resuspended in neurobasal medium. We showed that the osteoclast-derived EV were able to promote axonal growth as depicted in Fig. 2G, supporting our previous observations. Under osteoclast-derived EV exposure, the number of axons to cross the microchannels was similar to the positive control, while the quantification of neurite’s length revealed a significant increase (Fig. 2H).

### Total osteoclast secretome and osteoclast-derived EV enriched fraction activate EGFR related signaling pathways on sensory neurons

The activity of different receptor tyrosine kinase (RTK) have been implicated in neuronal development, growth, survival and axonal regeneration [41]. To understand the mechanisms activated in the context of axonal outgrowth under total osteoclast secretome and EV-depleted stimuli, we determined the phosphorylation/activation level of RTK and downstream molecules in the DRG neurons.

#### EGFR family signaling pathway is involved in the axonal growth of DRG sensory neurons under osteoclasts secretome stimulation

**Fig. 3:**
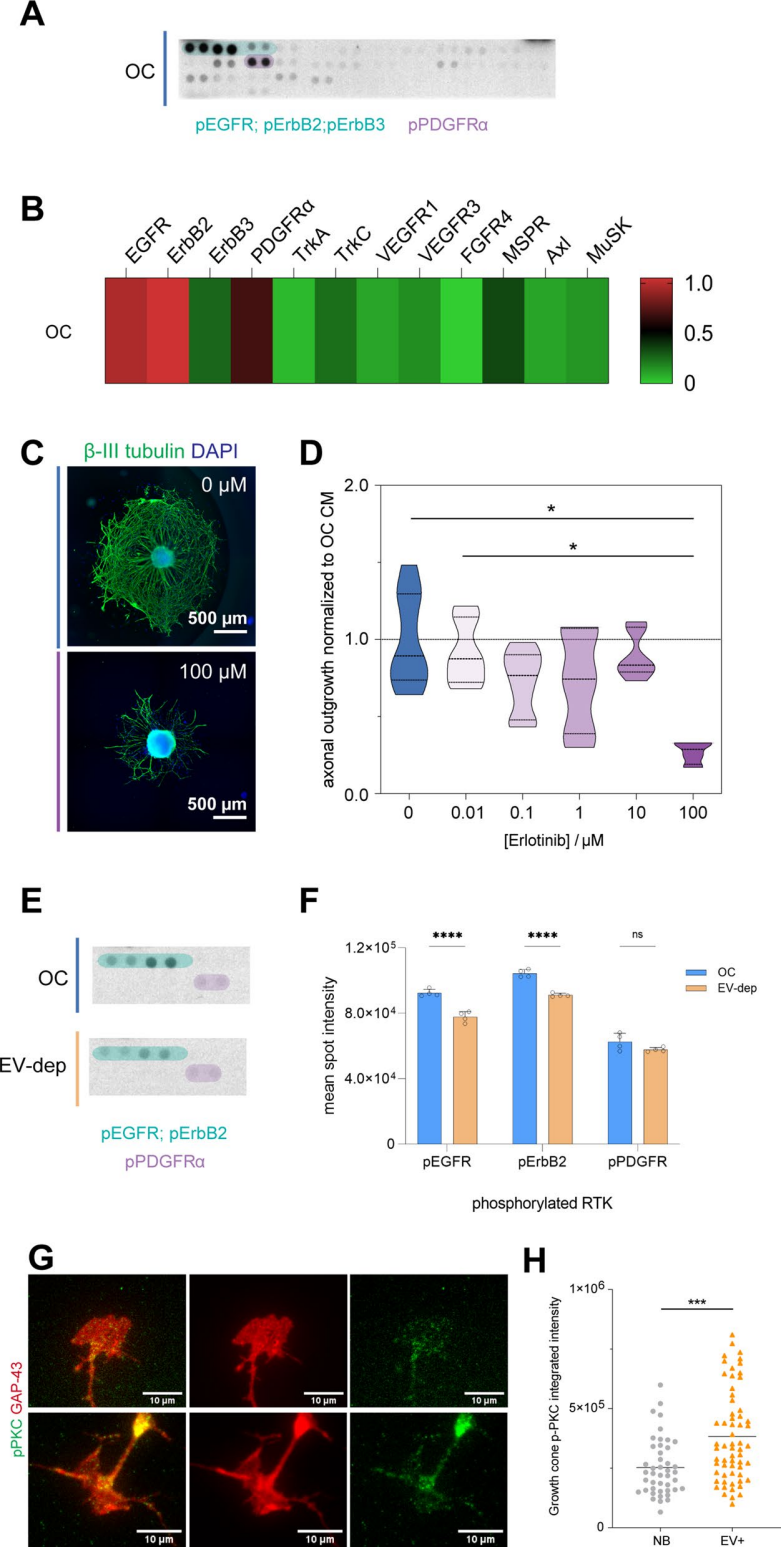
Epidermal-growth factor receptor (EGFR) activation. **A** Screening of receptor tyrosine kinases (RTK) phosphorylation levels in DRG cultures exposed to osteoclast secretome. Images of the X-ray films. For the analysis, 100 µg of protein lysate from 3 independent experiments (n = 3), was pooled. Elliptical shapes highlighting the spots corresponding to epidermal-growth factor receptors (EGFR, ErbB2 and ErbB3, light green) and platelet-derived growth factor receptor-alpha (PDGFα, light purple). **B** Heatmap representing the relative spot intensity for the activated receptors calculated from the pixel density, showing the primary activation of two different families: EGFR family and PDGF. **C** Pharmacological inhibition of EGFR and ErbB2 with increasing doses of Erlotininb. Representative images of DRG treated with different concentrations of Erlotinib for 72 h (βIII tubulin in green and nuclei in blue, scale bar 500 µm). **D** Quantification of axonal outgrowth of sensory neurons blocked with EGFR inhibitor—Erlotinib at different concentrations added to osteoclast conditioned medium. Data represented as violon plot *p ≤ 0.05. **E** Levels of receptor tyrosine kinases (RTK) phosphorylation in DRG cultures exposed to osteoclast secretome (OC, blue) and EV-depleted secretome (EV-dep, light orange). Images of the X-ray films. Elliptical shapes highlighting the spots corresponding to epidermal-growth factor receptors (EGFR, ErbB2 and ErbB3, light green) and platelet-derived growth factor receptor-alpha (PDGFα, light purple). **F** Graph representing the mean spot intensity of the activated receptors EGFR, ErbB2 and PDGFα for the DRG exposed to osteoclast secretome (OC, blue) and EV-depleted secretome (EV-dep, light orange). Data represented as bars with individual values (n = 4), mean ± SD, ns—non-significative; ****p ≤ 0.0001. **G** Representative images of sensory neurons growth cones exposed to neurobasal (NB; upper row) vs. EV enriched fraction (EV+; lower row), stained against growth-associated protein (GAP-43, red) and phosphorylated PKCα (green); scale bar: 10 µm. **H** Quantification of the integrated intensity of phosphorylated PKCα at the growth cones exposed to NB (grey) vs. EV+ (orange). Intensity of phosphorylated PKCα normalized for the growth cone area calculated through GAP-43 staining. Results are presented as scatter dot plot; ***p ≤ 0.01

DRG protein lysates exposed to osteoclast secretome were screened to quantify the phosphorylation level of over 30 RTK. An overview of the possible signaling pathways activated over osteoclast secretome stimuli was observed in the array (Fig. 3A). Epidermal growth factor receptor (EGFR), ErbB2, and platelet-derived growth factor receptor alpha (PDGFRα) displayed higher activation levels (Fig. 3A). Low levels of TrkA, TrkB (absent), and TrkC phosphorylation were observed (Fig. 3B), further confirming the low contribution of NGF, BDNF, NT-3, and NT-4/5 neurotrophins on the osteoclast mediated axonal growth. The activation of the ErbB2 receptor in DRG neurons could be triggered by heterodimerization with EGFR, since ErbB2 is an orphan receptor with no characterized ligand, which can be activated by spontaneous homodimer formation (in overexpressing cells) or by heterodimerization with another ligand-bound or EGF family transactivated receptor [42, 43]. In the context of nerve repair, these results are in agreement with the literature where ErbB receptors expression was shown to be increased in DRG upon lesion [44]. Therefore, osteoclasts might promote axonal outgrowth through EGFR family signaling, described to be involved in neuronal repair.

To correlate the contribution of EGFR and ErbB2 signaling on axonal outgrowth induced by osteoclasts secretome stimuli, receptor-mediated inhibition using pharmacological blockers was performed. Erlotinib is an EGFR inhibitor that reversibly binds to the intracellular tyrosine kinase domain of the receptor. Still, it has been shown to inhibit both EGFR and ErbB2 signaling pathways [43, 45–47]. Our results show that the neurotrophic effect of osteoclasts was reduced in the presence of the high Erlotinib concentration (Fig. 3C, D), without compromising the cell viability and metabolic activity (Additional file 1: Fig. S4), suggesting that both EGFR/EGFR homodimers and EGFR/ErbB2 heterodimers might contribute to the osteoclast-mediated effect in axonal outgrowth.

#### ErbB2 phosphorylation is reduced upon EV depletion while protein kinase C (PKC) phosphorylation is increased after EV exposure

To understand if EV depletion modulates the EGFR/ErbB2 phosphorylation levels, we evaluated the phosphorylation state of the EGFR family on DRG after exposure to EV-depleted osteoclasts secretome. Remarkably, a significant decrease in both EGFR and ErbB2 phosphorylation levels was observed in the absence of osteoclast-derived EV (Fig. 3E, F), supporting the contribution of this signaling pathway to the EV osteoclast-mediated axonal growth. No alterations in the activation levels of PDGFRα was observed upon DRG stimulation with EV-depleted secretome (Fig. 3F).

To strengthen our hypothesis on the involvement of osteoclast-derived EV in the activation of EGFR/ErbB2 signaling pathway in axonal growth, protein kinase C (PKC) phosphorylation levels were quantified at the growth cones in microfluidic devices.

To maximize our experimental readout, we allowed sensory axons to accumulate in the axonal compartment where we performed a starving period with plain neurobasal medium for 5 h. Afterward, terminals were stimulated for 10 min with osteoclasts-derived EV. The phosphorylation levels were normalized for the growth cone area stained for growth-associated protein-43 (GAP-43). PKC was shown to be preferentially stimulated by EGFR/ErbB2 heterodimers over Akt downstream pathway [48, 49]. We observed an accumulation of phosphorylated PKC at the growth cones stained for GAP-43. A significantly higher phosphorylation level was detected at the nerve terminals exposed to osteoclast-derived EV, as depicted in Fig. 3G, H.

To unravel whether the osteoclasts lineage was expressing ligands that could activate these signaling pathways, a personalized primePCR was designed targeting the EGF receptors family ligands. Gene expression was normalized for the GAPDH housekeeping gene, followed by a fold-change calculation relative to the pre-osteoclasts expression levels. The results indicate that the differentiated osteoclasts express higher amounts of heparin-binding EGF (Hb-EGF), while pre-osteoclasts express higher amounts of Neuregulin-4. Independently of the differentiation stage, both express Amphiregulin and Neuregulin 1 and 2 (Additional file 1: Fig. S5). Further proteomic analysis to confirm the presence of these proteins in the EV cargo will be a valuable input to the osteoclasts-DRG crosstalk.

### Osteoclast-derived EV are internalized by sensory neurons

**Fig. 4:**
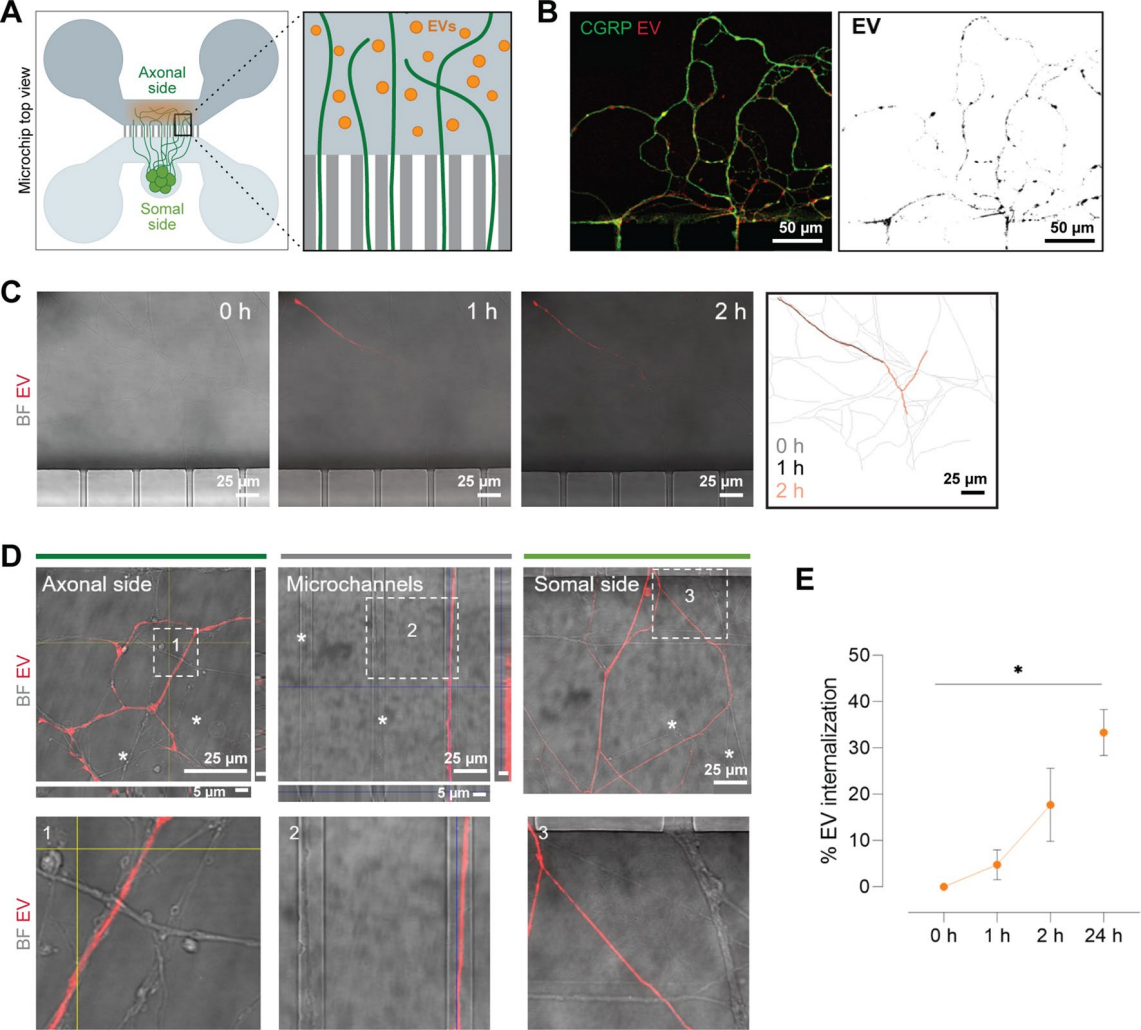
Internalization profile of osteoclast-derived EV by dorsal root ganglia (DRG) neurons in compartmentalized microfluidic chips. **A** Schematic representation of the experimental setup: microfluidic device with DRG culture on the somal side growing towards the axonal side. EV labelled with PKH26 lipophilic dye (red) added to the axonal side in close contact with nerve terminals. **B** Representative images of sensory neurons stained against calcitonin gene-related peptide (CGRP, green) with internalized EV (red) in the axonal compartment in the microfluidic devices; scale bar: 50 µm. **C** Representative images of EV labelled internalization by sensory neurons. Live imaging was performed under controlled temperature and CO_2_ over 0, 1 and 2 h showing the sensory neurons in brightfield (grey) and internalized EV (red); scale bar: 25 µm. Superimposed tracing of total neurites (grey) and neurites with internalized EV (black 1 h, orange 2 h), attained with Simple Neurite Tracer plugin, Fiji software, are shown in the far right. **D** Representative images of EV labelled internalization by sensory neurons after 24 h incubation. Images from axonal side, microchannels and somal side of the microfluidic device. Brightfield images showing the sensory neurons extensions and EV in red. White asterisks indicate sensory neurons without internalized EV. Orthogonal projections depicting the selective internalization of the EV (red); scale bar: 25 µm top view and 1 µm orthogonal view. Lower row shows zoom in of the indicated squares 1, 2 and 3. **E** Quantification of the EV internalization throughout time. Data represented as percentage of total neurites (mean ± SD), *p ≤ 0.05

Several studies describe how EV can interact with the recipient cell: by interacting with surface receptors at the nerve terminals, by fusing with neuronal cells membrane or by internalization [50]. Herein we labelled the osteoclast-derived EV (with lipophilic marker PKH26) and tracked the EV mobilization added to the axonal compartment in the microfluidic chips (Fig. 4A). We observed that the sensory neurons with internalized EV were positively stained for calcitonin gene-related peptide positive (Fig. 4B), characteristic of neuropeptidergic fibers. To understand the kinetics of interaction between the EV and sensory terminals, live imaging of EV internalization was performed over 2 h (controlled temperature and CO_2_). The uptake of the osteoclast-derived EV was observed after 45–60 min incubation (Fig. 4C). An increase in the fluorescence intensity, homogeneous distributed throughout the neurite extension, was observed with the increased incubation period (up to 2 h live, Fig. 4C). After 1 h incubation, 5% of the neurites had uptake EV, while after 2 h incubation the internalization almost reached 20% of the total fibers (Fig. 4E). For longer exposure periods, cells were kept at incubator and fixed after 24 h. EV positive signal was observed at the axonal, microchannels and somal compartments, inside the neurites, suggesting the anterograde transport of the vesicles towards the cell soma (Fig. 4D). After 24 h incubation the percentage of neurites loaded with EV reached one-third (33%) (Fig. 4E). Orto-projected and zoomed images of axonal side, microchannels and somal side enlighten the selective EV internalization within the sensory neurons in the same microfluidic device (Fig. 4D, unlabelled neurites marked with an asterisk). PKH26-positive EV only entered the neurons when the EV pellet was used, ruling out a transfer of excess dye. No free EV were detected at the somal compartment (Additional file 1: Fig. S6).

### Sensory neurons electrical activity is triggered by osteoclast secretome but not mediated by the EV

To unravel the electrophysiological implications of the axonal exposure to the osteoclast total secretome and osteoclast-derived EV, a combination of substrate-integrated microelectrode arrays (MEAs) with custom-made microfluidic chambers [51] were used. MEAs enable non-invasive, thus repeatable, recordings of extracellular action potentials. Although recordings of random DRG cultures have been previously demonstrated [52], these were not adapted to the study of peripheral innervation. Here, we employed microElectrode–microFluidic (µEF) devices, which allowed us to monitor axonal activity with high fidelity [51, 53].

**Fig. 5:**
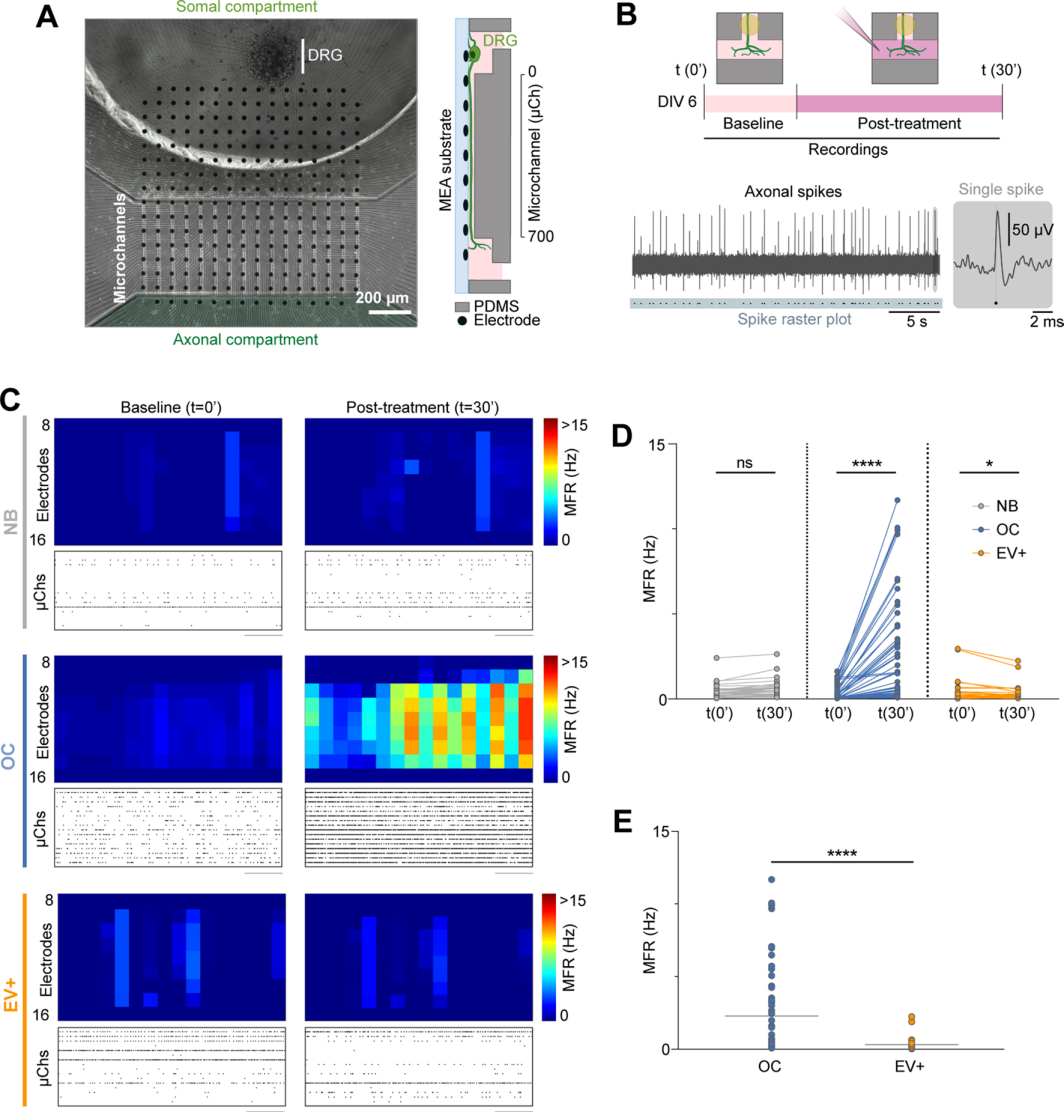
Electrophysiology studies on DRG neurons in microfluidic devices stimulated with osteoclasts secretome vs. osteoclast-derived EV. **A** Phase-contrast microscopy image mosaic of an organotypic dorsal root ganglia (DRG) culture at 6 days in vitro (DIV). The whole microelectrode array (MEA) (1.5 × 1.5 mm) active area is shown by a combination of 9 mosaic images (10× objective) from different parts of the culture. A PDMS device composed of 16 microchannels (10 μm width; 700 μm length) is aligned to encompass 7 microelectrodes. Details of axonal morphology can be seen in the somal compartment, microchannels and axonal compartment (scale bar 200 μm). A schematized version of a microchannel is shown on the right. **B** Electrophysiological trace of 30 s of baseline activity from an electrode (within a microchannel) at 6 DIV and corresponding spike raster plot. Inset shows a single spike. **C** Representative activity maps (microchannel area only, electrode rows 9–15) of baseline and post-treatment (time 30 min) activity for each condition (NB—neurobasal; OC—osteoclasts secretome; EV+—osteoclast-derived EV). Each pixel corresponds to an electrode and the mean firing rate (MFR) is color-coded. Representative raster plots of 60 s of activity are shown below each activity map. Each row corresponds to the spike raster plot from the central electrode of a single microchannel. **D** Before-after plot of every active microchannel after treatment. ns = not significant, *0.01 < p < 0.05, **0.001 < p < 0.01, ***p < 0.001, ****p < 0.0001. **E** Scatter dot plots of the active microchannels’ MFR at 30 min post-treatment (OC—osteoclasts secretome; EV+—osteoclast-derived EV). Data from 35 to 61 microchannels from 3 to 5 independent μEFs

Regardless of the explant position (outside or inside the array area, Fig. 5A), in most cases, we could only detect activity within the microchannels. This allowed us to directly compare baseline and post-treatment levels of axonal activity, as most axons within the microchannels are expected to have extended to the axonal compartment.

DRG terminals were exposed to two conditions comprising osteoclast secretome: (i) total osteoclast secretome (OC) and (ii) osteoclast-derived EV (EV+) and compared to NGF-containing neurobasal control (NB) (Fig. 5B).

Electrophysiology recordings show that the DRG exposed to osteoclasts secretome present an increased mean firing rate (MFR), when compared to the baseline recorded immediately before treatment (Fig. 5C). The control treatment, supplemented with NGF, does not produce a significant effect in the MFR. Curiously, the effect on the firing rate of the sensory neurons remained unchanged upon the addition of the osteoclasts EV enriched fraction (Fig. 5D, E). Different timepoints were tested, up to 24 h, but no alterations were detected (Additional file 1: Fig. S7). These results suggest that the sensory neurons electrophysiological activity is triggered by soluble factors present in the total osteoclasts secretome, however similar effect was not reproduced by the EV alone. The mechanisms supporting this effect require further elucidation.

## Discussion

Neuronal axonal growth is mediated by different classes of neurotrophic factors which include classic neurotrophins (e.g., NGF, BDNF, NT-3/4) [54], pro-inflammatory cytokines (e.g., Il-1β, TNF-α) [55–57] or other soluble molecules secreted by different cells in response to their surrounding microenvironment. Osteoclasts were shown to induce axonal growth by netrin-1, under inflammatory conditions [9, 10]. In our experimental set up, no NGF, BDNF, GDNF, neurotrophins nor netrins were found significantly expressed by osteoclast lineage. Still, the expression profile of these factors might completely change when osteoclasts are under pathological conditions such as inflammation, tumor, either simulated in vitro or in animal models.

During the last decade, emerging findings attributed to EV trading a central role in peripheral tissue innervation but in contexts of harsh conditions such tumor microenvironment and neuroinflammation [58]. As example, the neurotrophic-promoting activities of EV was demonstrated in pathological sensory axonogenesis in squamous cell carcinoma [59]. EV secreted by the cells at the tumoral microenvironment enhance sensory innervation through EphrinB1 guidance molecule and the pharmacological blockade of EV release attenuate the tumor innervation in vivo [59]. The physiological role of EV in establishing correct peripheral tissue innervation under homeostatic conditions and the mechanistic understanding of the EV mediated guiding of axonal projections is still poorly understood. Here we demonstrate that, under non-pathological conditions, osteoclasts-induced axonal growth is dependent on the secreted EV providing a new mechanism for the interplay between sensory terminals and bone resorbing cells. We confirmed these data by testing either the EV-depleted osteoclast secretome or the osteoclast-derived EV directly on DRG sensory neurons. The EV depletion from osteoclast secretome revealed a negative impact on axonal extension. The opposite effect was observed when osteoclast-derived EV enriched fraction was added to the axonal terminals, promoting and extensive axonal growth.

Zhang et.al. demonstrated that both cell soma and axons of cortical neurons were able to uptake the mesenchymal cells derived EV. The internalization was impaired by botulinum neurotoxin showing the involvement of soluble *N*-ethylmaleimide-sensitive factor attachment protein receptor (SNARE) complex [20]. In our study, we were interested in the specific interaction of osteoclast-derived EV and the axonal terminals, since only the axonal projections are present at the bone microenvironment in close contact with bone cells and their secreted products [60]. Therefore, compartmentalized microfluidic devices were applied to culture sensory neurons, allowing spatial and fluidic separation of the cell soma from distal axons [1, 39], to evaluate the EV internalization process. We showed that the osteoclast-derived EV were up taken by the axonal terminals, revealing 5% internalization during the first hour and increasing to 30% after 24 h. Neurons were demonstrated to internalize EV by endocytosis with accumulation within endosome-like structures [15, 18]. Given the distribution of the fluorescence intensity inside the neuronal extensions, we suggest that similar mechanisms are taking place in our experimental setup. Interestingly, despite the homogeneity of the nerve fibers phenotype present in culture (CGRP positive fibers), the EV were not equally internalized by the sensory neurons, since after the 24 h exposure there were fibers completely clear from EV. Previous studies reported that DRG neurons selectively uptake EV from glial cells rather than EV from fibroblasts [14]. In our experimental setup the EV source is the same (osteoclasts cells), still the internalization process can be mediated by specific interactions between the EV cargo and receptors at the nerve fiber terminals. This remains an open question that need further investigation.

There is an extensive discussion in the literature concerning the direct involvement of EGFR activation/inhibition in axonal regeneration [61–65]. EGFR phosphorylation has been implicated in signaling inhibition of axonal growth in the central nervous system [61, 62]. At the periphery, Koprivica et al*.* showed that EGFR inhibitors effectively promoted neurite outgrowth from cultured DRG [66]. However, we and others previously demonstrated an increased expression of the EGFR family in DRG after lesion [44, 67, 68], suggesting a possible role on neuronal regeneration. In this study we demonstrate that EV-depleted osteoclast secretome produced not only a significant decrease in axonal growth but also a significative reduction in EGFR family phosphorylation. Our data indicate that EGFR signaling has a role in axonal outgrowth promoted by osteoclast secretome. EGFR inhibition with Erlotinib, described to inhibit both EGFR and ErbB2 receptor kinases [43, 46, 47], resulted in a significant reduction in the axonal outgrowth area. Our findings are consistent with prior observations showing that the phosphorylation of EGFR enhances neurite outgrowth [61, 69–74]. Our data strongly indicate that the osteoclast-derived EV activate similar mechanisms, at the axonal growth cone, as significant increase of PCKα phosphorylation was observed. In fact, EGFR/ErbB2 heterodimers were reported to preferentially stimulate PKC, whereas ErbB2/ErbB3 heterodimers preferentially stimulate Akt signaling pathway [48, 49]. In tumoral context, it was shown that the EV can incorporate in their cargos EGFR receptors or EGFR ligands to deliver to the recipient cells promoting metastases or inducing resistance in drug-sensitive cells [75–78]. Unraveling the osteoclast-derived EV cargo will further elucidate the mechanism behind the EGFR activation. Our results largely contribute to support the hypothesis that EGFR activation is associated with an enhancement of axonal growth.

Abnormal increase of sensory nerve fibers axonal growth was demonstrated in skeletal diseases. Sensory nerve fibers undergo a remarkable sprouting and pathological reorganization which drive the pain [12, 28–31]. In pathological scenarios, such as fracture, bone cancer, or osteoporosis there is an imbalance between bone formation and bone resorption, alterations in the innervation pattern are often observed, suggesting a dynamic crosstalk within the bone microenvironment [12, 28, 30, 79]. Neurotransmitters and axonal guidance cues have been shown to have an effect in bone cells, particularly in osteoclasts activity. Calcitonin gene-related peptide (CGRP) has been shown to suppress osteoclast maturation and activity in vitro [80], whereas substance P (SP) can drive RANKL-independent osteoclastogenesis [81]. Semaphorin 3A (Sema3a) is a vital axonal guidance cue which has been shown to inhibit osteoclastogenesis and promote osteoblast differentiation [6]. Cells at the bone microenvironment release mediators responsible for activation of sensory nerves triggering electrical signal propagation towards central pathways, thus evoking pain [82, 83]. To understand if osteoclasts, under physiological conditions, induce the electrical signal propagation on sensory neurons, we measured the electrophysiological activity levels upon stimuli with the osteoclast secretome. We employed microElectrode–microFluidic (µEF) devices, to precisely expose only the nerve terminals to the stimuli, while recording the electrical propagation through the neuronal extensions towards the cell soma. Unlike central nervous system neurons in culture (e.g., hippocampal neurons), DRG neurons did not fire in bursts but rather exhibited a baseline activity with sporadic spontaneous spiking. Under normal conditions, this relatively low level of spontaneous activity also occurs in vivo [84]. The greater effect observed in the increasing of the MFR was related to the secretome from mature osteoclasts. The firing rate of the sensory neurons remained unchanged upon the addition of the osteoclasts EV enriched fraction. These results suggest that the sensory neurons electrophysiological activity is triggered by soluble factors present in the total osteoclasts secretome reflecting a possible pathway to be addressed to understand nerve sensitization on bone microenvironment. Stimulation of cortical neurons with glial EV were shown to increase the number of action potentials with unaltered spike amplitude [85]. Yet, similar effect was not reproduced in the sensory neurons through the stimulation with osteoclast-EV alone. This observation can indicate that osteoclast-derived EV are associated with sensory neurons extension but not directly with their neuronal activity. It would be relevant to collect the osteoclast-derived EV from bone microenvironment of inflammatory or metastatic mouse models and elucidate the role of EV on electrical signaling activation and propagation, related to nociception/pain since EV cargo is modified depending on the cellular and microenvironmental factors.

Overall, our study provides a new mechanism for sensory nerve growth mediated by bone resorbing cells—osteoclasts. We demonstrated that this effect is dependent on the EV released by these cells and achieved by targering EGFR/ErbB2 signaling/protein kinase C phosphorylation in sensory neurons. Our data also indicate that osteocalsts promote neuronal firing rate electrical activity in sensory neurons, but this effect is EV independent.

## Materials and methods

### Animals

All animal procedures were approved by the i3S ethics committee and by the Portuguese Agency for Animal Welfare (*Direção-Geral de Alimentação e Veterinária*) in accordance with the EU Directive (2010/63/EU) and Portuguese law (DL 113/2013). Mice were housed at 22 °C with a 12 h light/dark cycle with ad libitum access to water and food. Adult C57Bl/6 male mice (7 weeks-old) and pregnant females were sacrificed in a carbon dioxide chamber to obtain the primary cells (bone marrow, osteoclast lineage and sensory neurons).

### Bone marrow cells culture

Bone marrow stromal cells (BMSC) were isolated from tibiae and femur by flushing the bone marrow with α-MEM (Gibco, Thermo Fisher Scientific, Waltham, MA, USA) containing 10% (v/v) heat-inactivated (30 min at 56 °C) fetal bovine serum (FBS, Gibco, Thermo Fisher Scientific) and 1% (v/v) penicillin/streptomycin (P/S, Gibco, Thermo Fisher Scientific). Cells were plated in 75 cm^2^ tissue culture flasks. Non-adherent cells were removed after 3 days, and fresh medium was added. Cells were expanded from the colony-forming units for 1 week. Afterward, cells were detached with trypsin and seeded into 48 well plates at 5 × 10^4^ cells/cm^2^ density. No differentiation factors were added. The conditioned medium was collected after 24 h, centrifuged at 140 g, 4 °C, 5 min, and stored at − 80 °C. The conditioned medium was divided into small aliquots (500 µL–1 mL) before freezing to avoid repeated freeze/thaw cycles.

### Osteoclasts culture

Bone marrow cells were isolated from tibiae and femur by flushing the bone marrow with α-MEM containing 10% (v/v) FBS and 1% (v/v) P/S. To generate primary osteoclast precursors, the bone marrow mononuclear cell suspension was treated with red blood cells lysis buffer (ACK lysing buffer, #A1049201, Gibco, Thermo Fisher Scientific) for 1 min at room temperature (RT) and, after centrifugation, cells were plated in 10 cm diameter Petri dishes with 10 ng/mL macrophage colony-stimulating factor (M-CSF, PeproTech, London, UK) for 24 h. Afterward, the M-CSF concentration was increased to 30 ng/mL for an additional 3 days. Adherent cells were then detached with a cell scraper and seeded at a density of 5 × 10^4^ cells/cm^2^ (in 48 well plate, 1 mL of medium per well) in the presence of 30 ng/mL M-CSF alone or 30 ng/mL M-CSF and 100 ng/mL receptor activator of nuclear factor kappa-B ligand (RANKL, PeproTech) [86, 87]. Conditioned medium from pre-osteoclasts (M-CSF only) was collected after 24 h, centrifuged at 140 g, 4 °C, 5 min, and stored at − 80 °C. The cells exposed to both M-CSF and RANKL had the medium renewed at day 3 of culture, which was then collected after 24 h, corresponding to the mature osteoclast condition.

### qRT-PCR analysis

Total RNA was extracted using the Direct-zol™ RNA miniPrep according to the manufacturer’s protocol (Zymo Research). RNA final concentration and purity (OD260/280) was determined using a NanoDrop 2000 instrument (NanoDrop Technologies). RNA was reverse transcribed into cDNA using the NZY First-Strand cDNA Synthesis Kit (NZYTech), according to the manufacturer’s protocol. For the analysis of neurotrophins expression levels, a personalized PrimePCR array (Bio-Rad Laboratories) was performed. qRT-PCR experiments were run using an iCycler iQ5 PCR thermal cycler (Bio-Rad Laboratories) and analyzed with the iCycler IQTM software (Bio-Rad). Target gene expression was quantified using the cycle threshold (Ct) values and relative mRNA expression levels were calculated as follows: 2^(Ct reference gene − Ct target gene). GAPDH was used as a reference gene. Both target and reference genes were amplified with efficiencies between 100% ± 5%.

### Extracellular vesicles (EV) depletion from the osteoclast secretome and characterization

Osteoclasts were isolated and differentiated, as described in previous section. To obtain the supernatant for EV isolation, medium was prepared with EV-depleted FBS (obtained by ultracentrifugation). Cells were cultured with standard culture medium with 10% FBS, which was replaced by EV-depleted 1% FBS 24 h prior medium collection. All steps for the EV depletion were conducted under sterile conditions and in line with the published *Minimal information for studies of extracellular vesicles 2018* guidelines [88] and as elsewhere described [89]. Briefly, the secretome was collected, centrifuged 1000×*g* for 10 min to clear the cell debris, 2000×*g* for 10 min, followed by 10,000×*g* for 30 min. The supernatant was then ultracentrifuged at 100,000×*g* using 70Ti rotor (Beckman Coulter Genomics) for 120 min. The pellet containing exosomes was then washed with filtered PBS, ultracentrifuged overnight, and then stored at − 80 °C. All centrifugation steps were performed at 4 °C [90]. The supernatant was stored − 80 °C to perform the experiments comprising the EV-depleted secretome.

Western blot (WB) analysis was performed to the EV enriched fraction. Protein was quantified by DC Protein Assay kit (Bio-Rad). The same amount of protein (25 μg) of the EV enriched fraction were prepared in non-reducing loading buffer, denatured at 95 °C for 5 min, and loaded in 8% polyacrylamide SDS-PAGE gels. Resolved proteins were wet-transferred to nitrocellulose membranes, and membranes blocked with non-fat dry milk 5% solution. Membranes were probed overnight at 4 °C with hamster anti-mouse CD81 antibody [1:1000, clone Eat2 (MCA1846GA, Bio-Rad)]; rabbit anti-CD9 (1:1000, EXOAb-CD9A-1); rabbit anti-CD63 (1:1000, EXOAb-CD63A-1) and mouse anti-cytochrome C (7H8) (1:200, Santa Cruz Biotechnology). Membranes were probed with HRP-conjugated secondary antibody (GE Healthcare), incubated with ECL substrate. Chemiluminescence signal was detected using ChemiDoc (Bio-Rad) or with autoradiographic films (all from GE Healthcare) latter scanned on a GS-800 calibrated imaging densitometer (Bio-Rad).

The osteoclast-derived EV enriched fraction was further characterized by nanoparticle tracking analysis (NTA) and transmission electron microscopy (TEM), as previously described [91]. Briefly, for size and particle concentration evaluation, EV suspensions were diluted 1:500 in filtered PBS 1× and analyzed by NTA in a NanoSight NS300 device with NTA3.0 software. For the TEM negative staining, 10 µL of samples were mounted on Formvar/carbon film-coated mesh nickel grids (Electron Microscopy Sciences, Hatfield, PA, USA) and left standing for 2 min. The liquid in excess was removed with filter paper, and 10 µL of 1% uranyl acetate were added on to the grids and left standing for 10 s, after which liquid in excess was removed with filter paper. Visualization was carried out on a JEOL JEM 1400 TEM at 120 kV (Tokyo, Japan). Images were digitally recorded using a CCD digital camera Orious 1100 W (Tokyo, Japan) at the i3S Scientific Platforms Histology and Electron Microscopy.

### Dorsal root ganglia (DRG) culture

Embryonic DRG were obtained from 16.5 days-old (E16.5) C57BL/6 murine embryos, harvested and maintained on ice-cold Hank’s balanced salt solution (HBSS, Invitrogen). Ganglia were accessed through the dorsal side of the embryo after spinal cord removal. The meninges were cleaned, lumbar DRG from the L1 to L6 level were dissected and the roots were cut. The ganglia were kept in cold HBSS until seeding.

- *Organotypic cultures.* DRG were seeded into the lower wells of a 15-well µ-Slide Angiogenesis plate (#81506, Ibidi). Fibrin hydrogels were used to provide structural support for DRG culture. Hydrogels were formed by applying equal volumes of a solution of plasminogen-free fibrinogen, pooled from human plasma, and a thrombin solution containing CaCl_2_ and aprotinin [final concentration of fibrin components: 6 mg/mL fibrinogen (Sigma-Aldrich); 2 NIH U/mL thrombin from human plasma (Sigma-Aldrich); 2.5 mM CaCl_2_ (Sigma-Aldrich); 10 μg/mL aprotinin (Sigma-Aldrich)]. Before being used in the preparation of fibrin gels, fibrinogen was dissolved in ultrapure water, dialyzed against tris-buffered saline (TBS, pH 7.4), sterile-filtered and diluted to 12 mg/mL with sterile TBS. The fibrin gel polymerized for 15 min at 37 °C in a 5% CO_2_ humidified incubator, before the addition of culture media. DRG were cultured with neurobasal medium supplemented with 2% v/v B-27 Serum-Free Supplement® (B-27, Invitrogen), 60 µM 5-fluoro-2′-deoxyuridine (FDU, Sigma-Aldrich), 25 mM glucose (Sigma-Aldrich), 1 mM pyruvate (Sigma-Aldrich), 50 ng/mL 7S NGF (Calbiochem, Merck Millipore), 2 mM glutamine (BioWittacker, Lonza) and 1% P/S. Embryonic DRG explant cultures were left undisturbed for 24 h. Afterward, neurobasal media was replaced by the conditioned media collected from osteoclast cultures at different stages of differentiation (200 µg of total protein/well) for an additional 3 days (total protein concentration present in the different secretome was quantified, Additional file 1: Fig. S8). Controls with neurobasal supplemented with NGF and α-MEM supplemented with M-CSF and RANKL (no contact with cells) were performed. To assess the impact of EV depletion on axonal growth, DRG organotypic cultures were exposed to EV-depleted osteoclast secretome.
- *Axonal-specific exposure in microfluidic devices.* Commercially available microfluidic devices (Merck Millipore and Xona Microfluidics) were adapted for explant DRG culture and assembled, as previously described [67], on top of glass slides coated with 0.1 mg/mL poly-d-Lysine (PDL, Sigma-Aldrich) at 37 °C and 5 μg/mL laminin (Sigma-Aldrich). Cultures were left undisturbed for 24 h. At this time, the medium from the axonal side was substituted by total osteoclast secretome or EV-depleted secretome. To assess the effect of the osteoclast-derived EV enriched fraction on axonal growth, EV were resuspended in neurobasal medium at a concentration of 10^11^ EV/mL, corresponding to the initial EV concentration in the total secretome. DRG culture was left undisturbed for additional 72 h. A higher volume on the somal side was set to induce a slow net flow of liquid from the somal to the axonal compartment, thus ensuring that the conditioned medium was restricted to the axonal compartment.

### Quantification of axonal growth

Axonal outgrowth was quantified after 72 h of treatment with EV, EV-depleted secretome, total secretome and controls. The embryonic DRG samples (3D organotypic cultures and DRG on microfluidic devices) were fixed with 2% paraformaldehyde (PFA) in PBS for 10 min, followed by 10 min at 37 °C with 4% of PFA in PBS with 4% sucrose. Ganglia were permeabilized with 0.25% (v/v) Triton X-100 in PBS and incubated, for 30 min at RT, with blocking solution composed of 5% v/v normal goat serum (Invitrogen) and 5% v/v FBS in PBS. Samples were incubated with an antibody directed against the neuronal-specific marker βIII tubulin (Promega, United States) diluted 1:2000 in blocking solution, overnight at 4 °C. Afterward, cells were washed and incubated for 1 h at RT with the secondary antibody (Alexafluor 488/568, Invitrogen). Images were captured either with a confocal laser scanning microscope (CLSM) Leica TCS-SP5 AOBS equipped with LAS AF software (Leica Microsystems, Germany), at the i3S Bioimaging Platform, or with an IN Cell Analyzer 2000 equipped with IN Cell Investigator software (GE Healthcare, UK), at the i3S BioSciences Screening Platform. Organotypic cultures radial outgrowth, defined as the area between the ganglion edge and the outgrowth front, was determined. The outgrowth area was computed, according to Bessa et al*.* [92]*.* To quantify axonal outgrowth in microfluidic platforms, neurite outgrowth was measured with AxoFluidic, an algorithm designed to quantify neurite projection within these platforms [39]. The data were given by the spatial dependence decay function $f(x) = A \cdot \exp ( - x/\lambda )$ of the axons that can effectively cross the microchannels, where the constant *A* represents the number of axons that enter in the axonal compartment, and *λ* the scale of spatial decay, as a measure to represent the length of the neurites.

### Analysis of phospho-receptor tyrosine kinase (RTK) activation

A proteome profiler mouse phospho-RTK array kit (#ARY014, R&D system, USA) was used to quantify the phosphorylation level of 39 RTKs. After 72 h of treatment with conditioned medium and controls, the protein lysate of DRG was quantified and analyzed. According to the manufacturer’s instructions, for the array analysis, the same amount of protein was added to each membrane (100 µg). Each array membrane was exposed to X-ray film using a chemiluminescence detection system (Amersham, GE Healthcare). The film was scanned using Molecular Imager GS800 calibrated densitometer (Bio-Rad, Hercules, USA), and pixel density was quantified using Quantity One 1-D Analysis Software, v 4.6 (Bio-Rad). The results were presented as the mean spot intensity, which corresponds to the mean of the two spots for each receptor within the same membrane array.

### Pharmacological inhibition of epidermal growth factor receptor (EGFR) and ErbB2

Embryonic DRG were cultured in 15-well slides for 24 h. Erlotinib, an EGFR and ErbB2 inhibitor [47], was added to the conditioned medium at 10 nM, 100 nM, 1 µM, 10 µM, and 100 µM and tested on DRG cultures during 72 h. Afterward, axonal outgrowth and cell viability (Additional file 1) were measured.

### Quantification of protein kinase C (PKC) phosphorylation

To assess the phosphorylation status of PKCα in growth cones, the DRG were cultured in microfluidic devices for 72 h for axons to accumulate in the axonal compartment [1]. At this time point, a starving period was performed only in the axonal compartment with plain neurobasal medium for 5 h. Throughout the starving period, a volume difference between the axonal compartment and the somal compartment was maintained to prevent the diffusion of the complete medium from the somal to the axonal side. Axons were stimulated for 10 min with EV in neurobasal without NGF at a concentration of 10^11^ EV/mL, corresponding to the initial EV concentration in the total secretome, and immediately fixed afterward.

PKCα phosphorylation at the growth cones was performed by incubating DRG with primary antibodies directed against the growth-associated protein-43 [GAP-43 (Abcam)], and p-PKCα (Santa Cruz Biotechnology) diluted 1:1000 and 1:250, respectively, in blocking solution, overnight at 4 °C. Afterward, cells were washed and incubated for 1 h at RT with the secondary antibodies (Invitrogen) diluted 1:1000, in blocking solution. Images were captured with a widefield inverted microscope DMI6000 FFW (Leica Microsystems) equipped with LAS X software (Leica Microsystems) at the i3S Advanced Light Microscopy Platform. Growth cones were randomly chosen, based on GAP-43 fluorescence, without observation p-PKCα intensity. Total p-PKCα fluorescence was measured with Image J software, and the background intensity of each image was subtracted. For each selected growth cone, we determined the total of GAP-43 and p-PKCα fluorescence per area.

### EV labelling and internalization assay

Osteoclast-derived EV (or PBS as negative control) were labelled with PKH26 0.5 μM dye (Sigma-Aldrich), for 5 min at RT, and washed in VivaSpin® centrifugal columns (10 kDa cut-off). Labelled EV were added to the axonal compartment of DRG in microfluidic devices, at the same concentration present in the total osteoclast secretome (10^11^ EV/mL). Internalization was followed live for 120 min at laser scanning confocal microscopy (Leica TCS-SP5 AOBS) with controlled environment (temperature and CO_2_). Samples were fixed and analyzed after 24 h exposure.

DRG exposed to osteoclast-derived EV were stained against calcitonin-gene related peptide (CGRP). Briefly, after fixation, permeabilization and blocking as previous mentioned, cells were incubated with the primary antibody directed against CGRP (Sigma-Aldrich) diluted 1:8000, in blocking solution, overnight at 4 °C. Afterward, cells were washed and incubated for 1 h at RT with the secondary antibody (Alexa Fluor 488, Invitrogen) diluted 1:1000, in blocking solution. Images were acquired at laser scanning confocal microscopy (Leica TCS-SP5 AOBS). To quantify the percentage of EV internalization, neurites were semi-automatically traced with simple neurite tracer plug in for Image J software.

### Microelectrode–microfluidic cultures and electrophysiology recordings

The microfluidic devices (molds provided by INESC) were fabricated by mixing the polydimethylsiloxane (PDMS) elastomer (Sylgard® 184, DowCorning) with a curing agent (10:1, w/w), degassed and cured over the mold at 65 °C for 2 h. Custom microElectrode–microFluidic (µEF) devices were prepared as described previously [51]. Briefly, PDMS microfluidic chambers were aligned on top of microelectrode array (MEA) chips (MultiChannel Systems MCS GmbH, Germany), with 252 recording electrodes (30 µm in diameter and pitch of 100 µm) organized in a 16 × 16 grid. Microfluidic chambers had an appropriate microchannel spacing for compartmentalization and probing of axonal activity. Microfluidic chambers were also adapted by adding an extra smaller reservoir (Ø 3 mm), which allowed the seeding of the DRG in a central position to the electrode matrix [39]. µEF devices were composed of two separate compartments connected by 16 microchannels of 700 μm length × 9.6 μm height × 10 μm width. Each microchannel was probed with 7 electrodes, thus every axon extending to the axonal compartment was electrophysiologically monitored. After mounting, µEFs were sequentially coated with PDL (0.01 mg/mL) and laminin (5 μg/mL). The unbound laminin was aspirated, and chambers were refilled with complete neurobasal medium and left to equilibrate at 37 °C. Isolated embryonic DRG explants were placed and cultured as described before. DRG explants extended axons to the axonal compartment within the first 5 days in vitro (DIV). Then, treatments and recordings were performed at 6 DIV. This time point was chosen following preliminary studies that showed adequate electrophysiological maturation and culture viability at this stage [53]. Recordings at a sampling rate of 20 kHz were performed using a MEA2100 recording system (MCS GmbH, Germany). In every recording session, the temperature was maintained at 37 °C by an external temperature controller. After removing the cultures from the incubator, recordings only started after 5 min of habituation to avoid an effect due to mechanical perturbation. Then, a baseline recording (5 min) was obtained. Afterward, the medium from the axonal side was gently removed and replaced by 100 μL of treatment medium. The larger volume present on the somal compartment maintained a hydrodynamic pressure difference, inhibiting any flow from the axonal to the somal compartment.

Post-treatment recordings (30 min) were started as soon as the baseline stabilized following liquid flow perturbation (less than 1 min). Raw signals were high-pass filtered (200 Hz), and spikes were detected by a threshold set to 5xSD of the electrode noise. Spike data analysis was carried out in MATLAB R2018a (The MathWorks Inc., USA) using custom scripts. The mean firing rate (MFR) of each microchannel was calculated by averaging the MFR of the 5 inner electrodes (typically electrode rows 10–14), due to their superior signal-to-noise ratio. Microchannels with an MFR of at least 0.1 Hz in a given time point at 6 DIV were considered as active and included in the analysis.

### Statistical analysis

All experiments were run in triplicate and repeated at least 3 times. Data analysis was performed using GraphPad Prism 8.2.0 for Windows (GraphPad Software, San Diego CA, USA). Normality of the data was assessed. Statistical differences between groups were calculated using one-way analysis of variance, more precisely, the Kruskal–Wallis test followed by Dunns post-test for multiple comparisons for non-parametric distributions and One-way ANOVA for normal distributions. The non-parametric Mann–Whitney t-test was used to identify statistical differences when only two groups where being compared. Differences between groups were considered statistically significant when *0.01 < p < 0.05, **0.001 < p < 0.01, ***p < 0.001, ****p < 0.0001.

## Supplementary Information

**Additional file 1: Figure S1.** Evaluation of osteoclast differentiation. **Figure S2.** Dorsal root ganglia axonal growth. **Figure S3.** Quantification of secretome neurotrophins by enzyme-linked immunosorbent assay (ELISA). **Figure S4.** Drug toxicity assay—EGFR inhibitor. **Figure S5.** Genetic expression of EGFR/ErbB2 ligands by osteoclasts. **Figure S6.** PKH26 stained extracellular vesicles (EV) added to the axonal side in the microfluidic devices. **Figure S7.** Sensory neurons electrophysiological activity. **Figure S8.** Total protein quantification on the conditioned medium collected from different cell culture settings.

## Data Availability

All the data supporting the findings of this study are available from the corresponding author EN upon reasonable request.
