# Supplementary material for: Osteoclast-derived extracellular vesicles are implicated in sensory neurons sprouting through the activation of epidermal growth factor signaling
Source: Cell Biosci. 2022 Aug 14;12:127. doi: 10.1186/s13578-022-00864-w (PMC9375906; doi:10.1186/s13578-022-00864-w)
Supplement: Supplementary file 1 — Additional file 1: Figure S1. Evaluation of osteoclast differentiation. Figure S2. Dorsal root ganglia axonal growth. Figure S3. Quantification of secretome neurotrophins by enzyme-linked immunosorbent assay (ELISA). Figure S4. Drug toxicity assay—EGFR inhibitor. Figure S5. Genetic expression of EGFR/ErbB2 ligands by osteoclasts. Figure S6. PKH26 stained extracellular vesicles (EV) added to the axonal side in the microfluidic devices. Figure S7. Sensory neurons electrophysiological activity. Figure S8. Total protein quantification on the conditioned medium collected from different cell culture settings. [file 13578_2022_864_MOESM1_ESM.docx]

**Osteoclast-derived extracellular vesicles are implicated in sensory neurons sprouting through the activation of epidermal growth factor signaling**

Estrela Neto^1, 2^, Luís Leitão^1, 2, 3^, José C. Mateus^1, 2, 3^, Daniela M. Sousa^1, 2^, Cecília J. Alves^1, 2^, Miguel Aroso^1, 2^, Ana C. Monteiro^1, 2^, Francisco Conceição^1, 2, 3^, Richard O. C. Oreffo^5^, Jonathan West^6^, Paulo Aguiar^1, 2^ and Meriem Lamghari^1, 2,^ *

**Additional file 1**

**Figure S1. Evaluation of osteoclast differentiation**

Evaluation of osteoclast differentiation was performed by qRT-PCR analysis. RNA was isolated and purified as previously described in (Neto *et al.*, 2017). Briefly, total RNA was extracted using the Direct-zol™ RNA miniPrep according to the manufacturer’s protocol (Zymo Research). RNA final concentration and purity (OD260/280) was determined using a NanoDrop 2000 instrument (NanoDrop Technologies). RNA was reverse transcribed into cDNA using the NZY First-Strand cDNA Synthesis Kit (NZYTech), according to the manufacturer’s protocol. mRNA expression of differentiation markers was performed using a costum designed PrimePCR array (Bio-Rad Laboratories). qRT-PCR experiments were run using an iCycler iQ5 PCR thermal cycler (Bio-Rad Laboratories) and analyzed with the iCycler IQTM software (Bio-Rad). Target gene expression was quantified using the cycle threshold (Ct) values and relative mRNA expression levels were calculated as follows: 2^(Ct reference gene− Ct target gene). GAPDH was used as a reference gene. Both target and reference genes were amplified with efficiencies between 100% ± 5%.

Immunocytochemistry was performed to assess the morphology of the differentiated osteoclasts and the possible presence of macrophages in culture. Briefly, cells were fixed with 4% paraformaldehyde (PFA, Merck Millipore, Kenilworth, NJ, USA) in PBS during 10 min followed by permeabilization with 0.25% (v/v) Triton X-100 (Sigma-Aldrich, St. Louis, MO, USA) in PBS and incubated, for 30 min at RT, with blocking solution composed of 1% (w/v) Bovine Serum Albumin (BSA, Sigma-Aldrich) in PBS for 30 min at RT to block antibody unspecific binding. The cells were incubated with macrophages specific marker – anti-F4/80 (#14-4801-85, eBioscience, Thermo Fisher Scientific), 1:250 in blocking solution overnight at 4 °C. Afterwards cells were washed with PBS and incubated with Alexafluor 488-Phalloidin (Life Technologies, Thermo Fisher Scientific) 1:100 in PBS for 30 minutes at RT in the dark. At the end of the incubation time, the cells were washed with PBS and mounted using Vectashield with DAPI (Vector Labs, Burlingame, California, USA). Images were captured using the Inverted Fluorescence Microscope (Zeiss AxioVert, Carl Zeiss, Oberkochen, Germany), equipped with AxioVision SE64 Rel. 4.8 software. TRAP staining was also performed using an Acid phosphatase, Leukocyte (TRAP) kit (Sigma-Aldrich) according to manufacturer’s instructions. Images were acquired using a stereomicroscope (SZX10, Olympus, Shinjuku, Tokyo, Japan) coupled to a digital camera (DP21, Olympus).

**
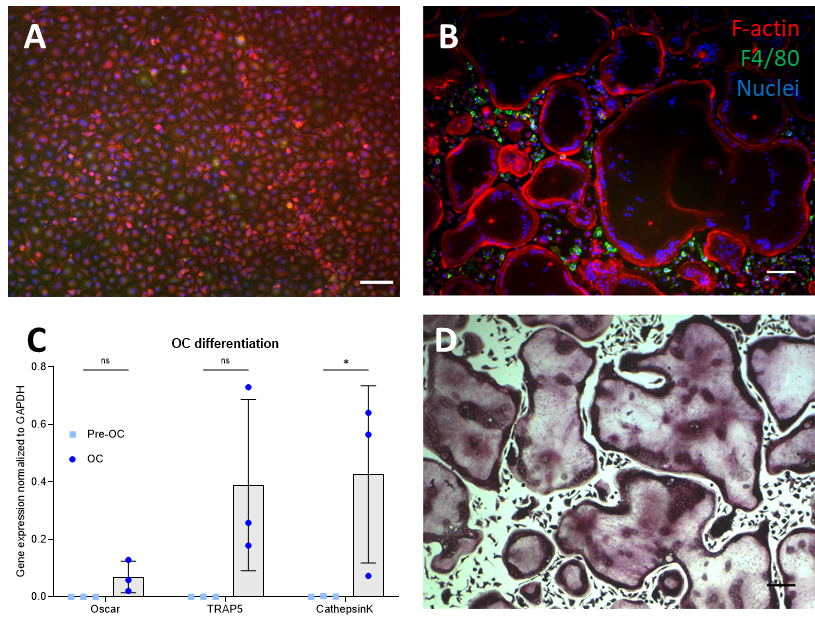
**

**Figure S1:** **Characterization of osteoclasts differentiation *in vitro.*** Osteoclasts were differentiated from mouse bone marrow hematopoietic lineage. **A.** Representative image of mononucleated pre-osteoclasts stained for f-action (red), nuclei (blue) and macrophages stained with F4/80 (green), cultured for 4 days with complete alpha-MEM supplemented with M-CSF. **B.** Image of mature osteoclasts stained for F-actin (red), nuclei (blue) and macrophages stained with F4/80 (green), revealing a large population of multinucleated osteoclasts with the low contribution of positive F4/80 macrophages present in the culture. **C.** Differentiation markers (osteoclast associated immunoglobulin-like receptor (OscAR), tartrate-resistant acid phosphatase (TRAP5) and cathepsin K (CTSK)) were evaluated by qPCR. Data represented as bars with individual values for 3 independent experiments, mean±SD. **D.** TRAP staining of mature osteoclasts differentiated *in vitro*. Scale bar 100 µm.

**Figure S2: Dorsal root ganglia axonal growth.**

To discard the possible effect of FBS (and enzymes present as matrix metalloproteinases (MMPs)) and even the effect of the cytokines used in osteoclast differentiation (M-CSF and RANKL in the osteoclasts condition), we exposed the DRG to the fresh medium without cell contact. DRG axonal outgrowth was quantified for ganglia exposed to alpha-MEM containing FBS and to the positive control of neurobasal supplemented with NGF and bone marrow stromal cells conditioned medium (BMSC) known to positively impact the axonal growth. There were no differences observed in the growth area regardless the medium formulation used.

**Figure S2**: **Sensory neurons** **axonal outgrowth area.** Automatic axonal outgrowth area quantification of DRG. DRG exposed to 1) non-conditioned osteoclast differentiation medium (OCm: alpha-MEM supplemented with 10% fetal bovine serum (FBS), receptor activator of nuclear factor kappa-Β ligand (RANKL) and macrophage colony-stimulating factor (M-CSF)); 2) standard medium composed of neurobasal supplemented with nerve growth factor (NB+NGF); 3) conditioned medium from bone marrow stromal cells (BMSC). Data represented as a violin plot; ns – non-significative.

**Figure S3: Quantification of secretome neurotrophins by enzyme-linked immunosorbent assay (ELISA)**

Conditioned media from osteoclasts and BMSC were concentrated 10 times by centrifugation at 4000 g for 45 min, using 3 kDa MW cut-off filter units (Merck Millipore). To detect and quantify the amount of NGF, BDNF, neurotrophin-3 (NT-3), and NT4/5 in the conditioned medium, a multi-neurotrophin rapid screening ELISA kit (#BEK-2231, Tebu-bio, France) was used according to the manufacturers’ protocol. Netrin-1 was quantified in the concentrated conditioned media using the ELISA development kit (EKC37454, Biomatik), also according to the manufacturer’s instructions.


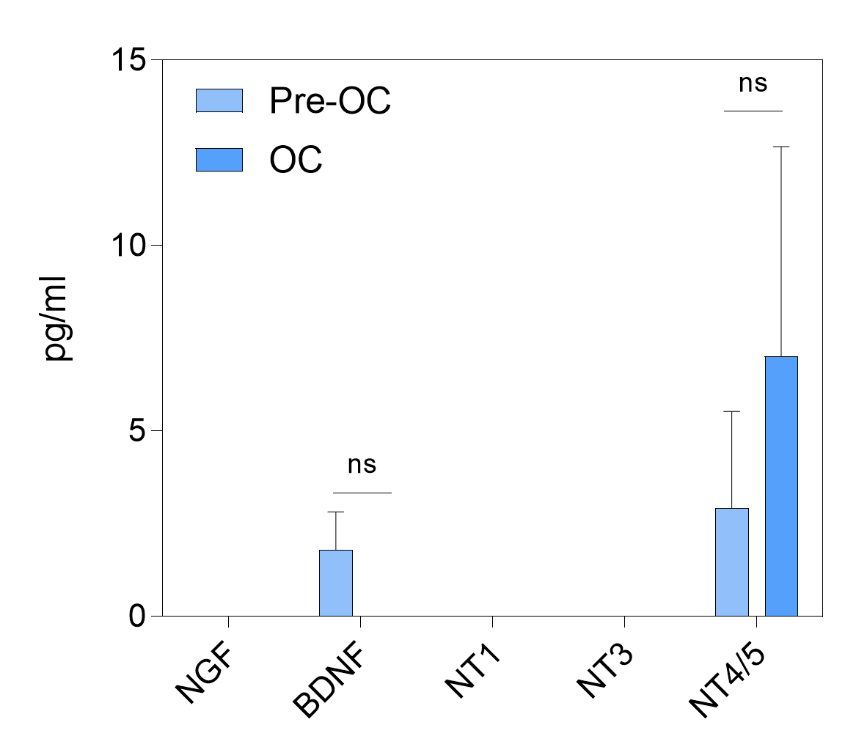


**Figure S3: Analysis of secreted neurotrophins by osteoclast lineage**. Quantification of nerve growth factor (NGF), brain-derived neurotrophic factor (BDNF), netrin-1, neurotrophin-3 (NT-3), and NT-4/5 by enzyme-linked immunosorbent assay (ELISA) on pre-osteoclasts and mature osteoclasts conditioned media. Data represented as mean±SD; ns – non-significative.

**Figure S4. Drug toxicity assay – EGFR inhibitor**

Cell viability assay was performed for the DRG treated with the highest concentration of Erlotinib to rule out the possible drug toxicity. Briefly, DRG were incubated with Calcein AM (Invitrogen) in PBS for 30 min at 37˚C. Calcein AM was washed out and DRG were incubated with propidium iodide (Sigma Aldrich) for 10 min at 37˚C. Images were acquired using IN Cell Analyzer 2000 equipped with IN Cell Investigator software. To address the DRG metabolic activity, resazurin solution (Sigma) was added to wells at a final concentration of 10% (v/v). After 4 h at 37 °C, 100 µL (pooled from two wells) were transferred to a 96-well black plate and fluorescence was measured (530 nm/590 nm) in a Spectra Max Gemini XS (Molecular Devices).


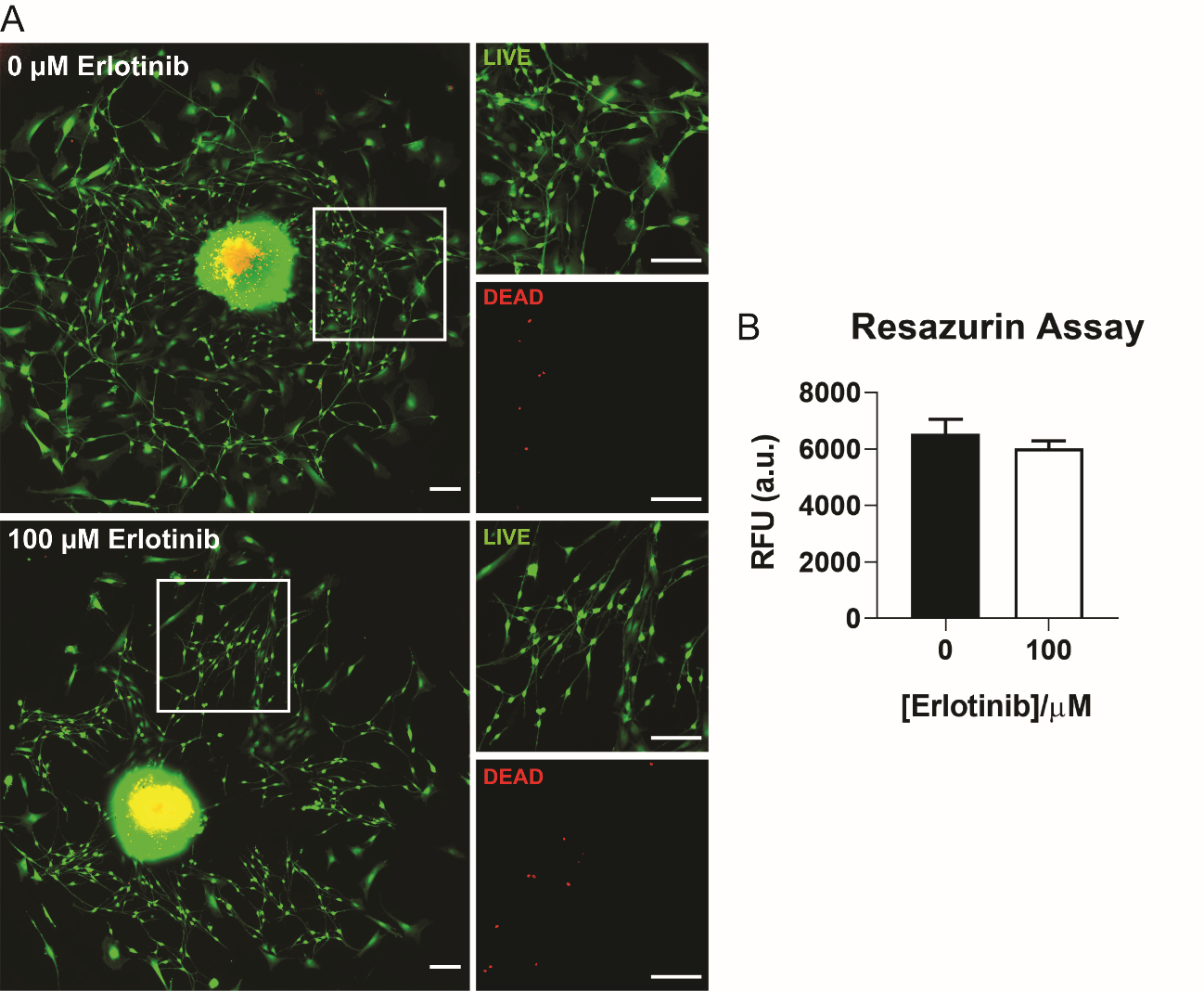


**Figure S4:** **Cell viability and metabolic activity of DRG upon treatment with 100 µM of Erlotinib*.*** **A.** Live/dead assay showing the live cells in green and dead cells in red for control conditions (neurobasal+NGF) and 100 µM of Erlotinib. Scale bar 100 µm. **B.** Evaluation of the metabolic activity of the DRG in control conditions (neurobasal+NGF) and 100 µM of Erlotinib. Data presented as relative fluorescence units (RFU).

**Figure S5: Genetic expression of EGFR/ErbB2 ligands by osteoclasts**

Gene expression of EGFR/ErbB2 ligands was evaluated on pre-osteoclasts and osteoclasts samples. A personalized primePCR was desinged to screen the osteoclasts gene expression targeting the EGF receptors family ligands. The gene expression was normalized for the GAPDH housekeeping gene and further fold change was calculated (relatively to the pre-OC gene expression).

**Figure S5: Genetic expression of EGFR/ErbB2 ligands by osteoclasts.** The EGFR family ligands are differently expressed by the osteoclasts dependent on the differentiation stage. Heparin-binding EGF (HB-EGF) is highly expressed by mature osteoclasts. Neuregulin-4 expression decreases with the osteoclast differentiation stage. Data represented as mean±SD, ***p≤0.01, ****p≤0.001.

**Figure S6. PKH26 stained extracellular vesicles (EV) added to the axonal side in the microfluidic devices**

Osteoclasts-derived EV (or PBS as negative control) were labelled with PKH26 0.5 μM dye (Sigma-Aldrich), for 5 min at RT, and washed in VivaSpin® centrifugal columns (10 kDa cut-off). Labelled EV were added to the axonal compartment of DRG in microfluidic devices, at the same concentration present in the total osteoclast secretome (10^11^ EV/mL). The larger volume present on the somal compartment maintained a hydrodynamic pressure difference, inhibiting any flow from the axonal to the somal compartment.


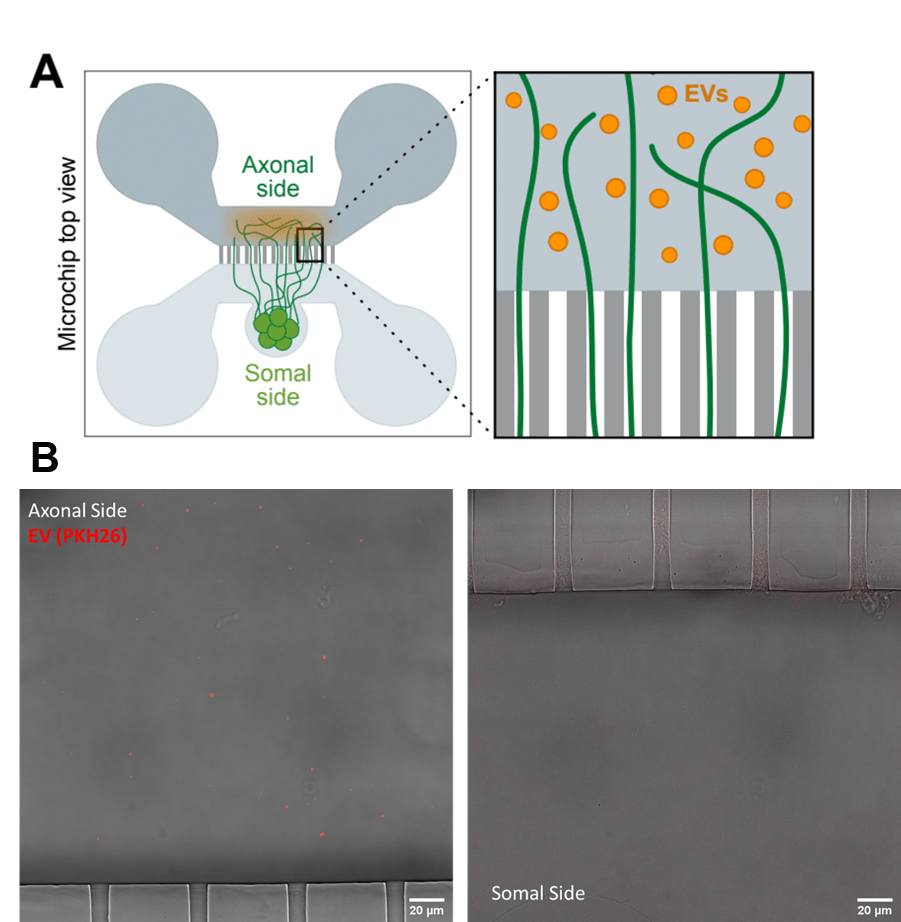


**Figure S6:** **Osteoclast-derived extracellular vesicles (EV) stained with PKH26 dye. A.** Schematic representation of the experimental setup. Microfluidic device with DRG culture on the somal side growing towards the axonal side. EV added to the axonal side in close contact with nerve terminals. **B.** Confocal images showing the presence of EV agglomerates in the axonal compartment. The fluidic isolation avoided the diffusion of the EV towards the somal side. Microfluidic features (microchannels) can be observed in brightfield and EV in red. Scale bar 20 µm.

**Figure S7. Sensory neurons electrophysiological activity**

Treatments and recordings were performed at 6 DIV of embryonic DRG culture. Recordings at a sampling rate of 20 kHz were performed using a MEA2100 recording system (MCS GmbH, Germany). A baseline recording (5 min) was obtained. Afterward, the medium from the axonal side was gently removed and replaced by 100 μl of treatment medium (neurobasal, osteoclast conditioned medium (OC) and osteoclast-derived EV (EV+).

Post-treatment (timepoint 30’) recordings were started as soon as the baseline stabilized following liquid flow perturbation (less than 1 min). Cultures were returned to the incubator until 3 hours after treatment when a post-treatment recording (timepoint 3h) of 5-10 min was performed. The day after, a final post-treatment recording (timepoint 24h) was performed for 5-10 min.

Raw signals were high-pass filtered (200 Hz), and spikes were detected by a threshold set to 5× SD of the electrode noise. Spike data analysis was carried out in MATLAB R2018a (The MathWorks Inc., USA) using custom scripts. The mean firing rate (MFR) of each microchannel was calculated by averaging the MFR of the 5 inner electrodes (typically electrode rows 10-14), due to their superior signal-to-noise ratio. Microchannels with an MFR of at least 0.1 Hz in a given time point at 6 DIV were considered as active and included in the analysis.


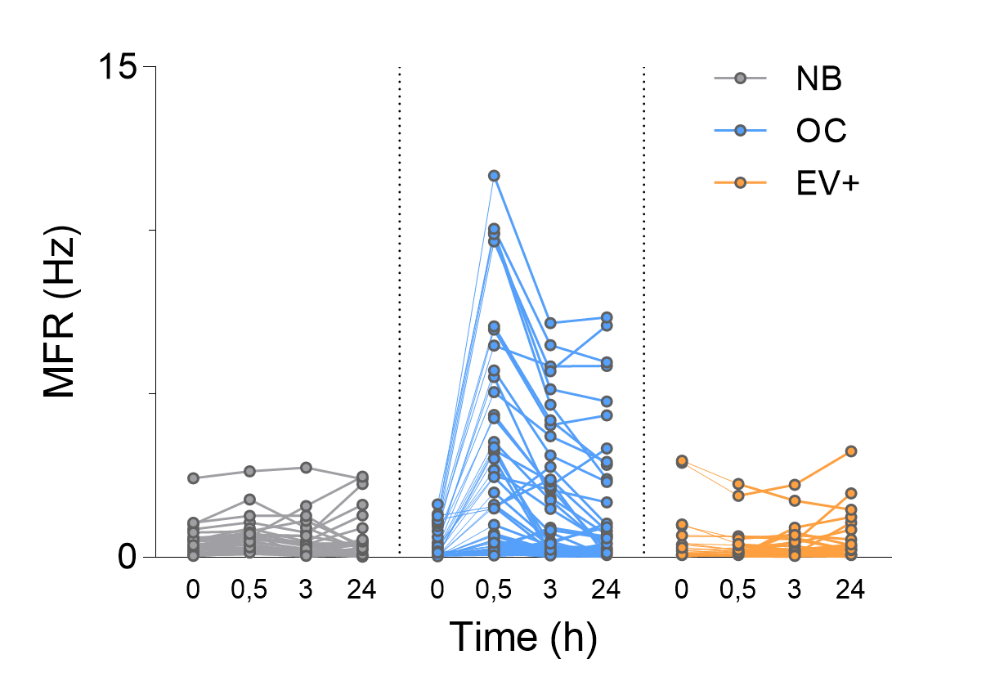


**Figure S7:** **Electrophysiology studies on dorsal root ganglia (DRG) neurons in microfluidic devices stimulated with osteoclasts secretome and osteoclast-derived extracellular vesicles (EV+).** MFR at baseline (0), 0,5h, 3h and 24h post-treatment. Data from 35-61 microchannels from 3-5 independent μEFs. No significant alterations were observed for the electrical activity of sensory neurons on neurobasal condition (NB) or stimulated with osteoclast-derived EV (EV+).

**Figure S8. Total protein quantification on the conditioned medium collected from different cell culture settings**

Upon collection of conditioned media from bone cells’ culture (BMSC, Pre-OC and OC), total amount of protein was quantified to expose the DRG cultures to a normalized amount of cell secreted factors.

**Figure S8: Quantification of total protein amount in the different secretome.** Prior adding the conditioned medium to the DRG cultures, the total amount of protein was quantified. Afterwards, for DRG stimulation, 200 ug of total protein from each condition was added to the DRG culture.

**References**

Neto, E. *et al.* (2017) ‘Axonal outgrowth, neuropeptides expression and receptors tyrosine kinase phosphorylation in 3D organotypic cultures of adult dorsal root ganglia’, *PLOS ONE*. Edited by Y. Tache, 12(7), p. e0181612. doi: 10.1371/journal.pone.0181612.
